# Right ventricular uncoupling in acute heart failure with preserved or mildly reduced ejection fraction: a simple parameter to predict long-term mortality

**DOI:** 10.47487/apcyccv.v6i2.466

**Published:** 2025-06-27

**Authors:** Lucrecia M. Burgos, Lucía Campos Cervera, María A. De Bortoli, Rocío C. Baro Vila, Franco N. Ballari, Mirta Diez

**Affiliations:** 1 Heart failure, pulmonary hypertension and heart transplant department. Instituto Cardiovascular de Buenos Aires. Buenos Aires, Argentina. Heart failure, pulmonary hypertension and heart transplant department Instituto Cardiovascular de Buenos Aires Buenos Aires Argentina; 2 Clinical cardiology department. Instituto Cardiovascular de Buenos Aires. Buenos Aires, Argentina. Clinical cardiology department Instituto Cardiovascular de Buenos Aires Buenos Aires Argentina

**Keywords:** Heart Failure, Prognosis, Pulmonary Hypertension, Insuficiencia Cardíaca, Pronóstico, Hipertensión Pulmonar

## Abstract

**Introduction.:**

Risk prediction in acute heart failure (AHF) has led to the development of multiple prognostic models. Emerging data highlight the prognostic significance of right ventricular (RV) to pulmonary artery (PA) uncoupling, which has been linked to adverse outcomes. Among patients with heart failure with preserved ejection fraction (HFpEF) and mildly reduced ejection fraction (HFmrEF), a highly heterogeneous group, the prognostic relevance of RV-PA uncoupling in forecasting long-term mortality is still not well defined. This study aimed to evaluate the association between RV-PA uncoupling and long-term mortality in a cohort of hospitalized HFpEF and HFmrEF patients.

**Materials and Methods.:**

We performed a retrospective analysis based on a prospective registry of adult patients admitted with a primary diagnosis of AHF between 2015 and 2020. Eligible patients had a left ventricular ejection fraction (LVEF) > 40%. The main outcome was all-cause mortality over long-term follow-up. RV-PA coupling was quantified using the ratio between tricuspid annular plane systolic excursion (TAPSE) and systolic pulmonary artery pressure (sPAP).

**Results.:**

Out of 465 patients, simultaneous estimation of TAPSE and sPAP was feasible in 361 cases (77%). During a median follow-up of 20.9 months, 100 patients (27.7%) died. A TAPSE/sPAP ratio of 0.38 was identified as the optimal cut-off for risk discrimination. Notably, 41.8% of patients had values below this threshold. Multivariable analysis confirmed that RV-PA uncoupling (TAPSE/sPAP < 0.38) was independently associated with increased long-term mortality (HR: 2.21; 95% CI 1.26-3.81; P = 0.005).

**Conclusion.:**

In patients hospitalized for AHF with preserved and mildly reduced ejection fraction, RV-PA uncoupling, as determined by the TAPSE/sPAP ratio, was independently associated with long-term all-cause mortality. This echocardiographic parameter may help identify a subgroup of patients at higher risk during follow-up.

## RESUMEN

**Introducción.:**

La estratificación del riesgo en la insuficiencia cardíaca aguda (ICA) ha dado lugar al desarrollo de múltiples modelos pronósticos. La evidencia reciente destaca la importancia del desacoplamiento entre el ventrículo derecho (VD) y la arteria pulmonar (AP), que se ha asociado con peores resultados clínicos. En los pacientes con insuficiencia cardíaca con fracción de eyección preservada (ICFEp) y levemente reducida (ICFElr), una población altamente heterogénea, el valor pronóstico del desacoplamiento VD-AP para predecir la mortalidad a largo plazo aún no está bien establecido. Este estudio tuvo como objetivo evaluar la asociación entre el acoplamiento VD-AP y la mortalidad a largo plazo en pacientes hospitalizados con ICFEp.

**Materiales y métodos.:**

Se realizó un análisis retrospectivo de una cohorte prospectiva de pacientes adultos internados entre 2015 y 2020 con diagnóstico principal de ICA y fracción de eyección del ventrículo izquierdo (FEVI) >40%. El objetivo primario fue la mortalidad por cualquier causa a largo plazo. El acoplamiento VD-AP se evaluó mediante la razón entre el desplazamiento sistólico del plano del anillo tricuspídeo (TAPSE) y la presión sistólica en la arteria pulmonar (sPAP).

**Resultados.:**

De los 465 pacientes, fue posible estimar simultáneamente TAPSE y sPAP en 361 casos (77%). Durante un seguimiento medio de 20,9 meses, se registraron 100 muertes (27,7%). La curva ROC identificó un valor óptimo de corte para TAPSE/sPAP de 0,38. El 41,8% de los pacientes presentó un cociente TAPSE/sPAP <0,38. El análisis multivariado confirmó que el desacoplamiento VD-AP (TAPSE/sPAP <0,38) se asoció de forma independiente con una mayor mortalidad a largo plazo (HR 2,21; IC 95%: 1,26-3,81; p=0,005).

**Conclusión.:**

En pacientes hospitalizados por ICA con fracción de eyección preservada y levemente reducida, el desacoplamiento VD-AP evaluado mediante la razón TAPSE/sPAP se asoció de manera independiente con la mortalidad por cualquier causa a largo plazo. Este parámetro ecocardiográfico puede ayudar a identificar un subgrupo de pacientes con mayor riesgo durante el seguimiento.

## Introduction

Heart failure (HF) with preserved ejection fraction (HFpEF) and mildly reduced ejection fraction (HFmrEF) are common and complex conditions. ^1^ Despite significant progress in the diagnosis and management of heart failure (HF), the overall prognosis remains poor and exhibits considerable heterogeneity across patient populations. ^2^ Consequently, the early identification of individuals with acute decompensated heart failure (AHF) who are at elevated risk of death is essential to guide individualized therapeutic strategies and optimize clinical outcomes. ^3^^,^^4^ Several validated, and clinically applicable risk stratification models have been developed to support decision-making in the management of patients with AHF. ^5^ Recently, there has been increasing evidence associating right ventricular (RV) dysfunction with higher morbidity and mortality in patients with HF, both with preserved and reduced systolic function. ^6^^-^^8^ It is present in approximately 4% to 50% of patients with HFpEF and HFmrEF, and this variability is partly due to the lack of consensus in its definitions. ^8^^,^^9^

Estimating RV function poses a challenge due to both anatomical and structural factors. The most commonly used parameters for estimation in clinical practice are tricuspid annular plane systolic excursion (TAPSE), fractional shortening, and systolic pulmonary artery pressure (sPAP) estimated by transthoracic echocardiography. The right ventricle to pulmonary artery (RV-AP), uncoupling measured through the relationship between TAPSE and sPAP, emerges as a novel parameter providing more information on the overall performance of the RV than each parameter alone. ^(10, 11)^ This relationship has been shown to be an independent prognostic factor for mortality in patients with HFpEF, ^(12, 13)^ with a cutoff point of <0.36 being established to predict a worse outcome. ^10^

Given that HFpEF and HFmrEF are heterogeneous conditions, identifying prognostic factors that allow us to stratify patients based on risk is appealing for achieving better outcomes. This study aimed to evaluate the prognostic value of the relationship between TAPSE and sPAP in hospitalized HFpEF and HFmrEF patients with AHF to predict short- and long-term mortality.

## Material and methods

### Study design and population

A single-center retrospective cohort study was conducted, utilizing prospectively collected data. The study included consecutively admitted adult patients at a specialized cardiovascular hospital with a primary diagnosis of acute heart failure (AHF) between January 2015 and January 2020, with an ejection fraction greater than 40%. Two cardiology specialists independently confirmed the diagnosis based on information obtained from patient history, physical examination, and complementary studies.

Patients with AHF secondary to acute myocardial infarction, severe sepsis, or pulmonary embolism were excluded. Additionally, patients who did not undergo an echocardiogram within three months before the index hospitalization or did not have TAPSE and/or sPAP measurements were also excluded.

### Endpoints

The primary endpoint was long-term all-cause mortality. Long-term mortality was assessed for patients discharged after the index hospitalization until their last available outpatient or inpatient assessment. Information on mortality was obtained from patients’ medical records.

### Data collection

Demographic information, comorbidities, admission physical examination, clinical presentation type, prior medical treatment, implemented therapy, clinical outcomes, and complementary test results were collected in an HF center database.

### Echocardiographic variables

The following echocardiographic variables were studied and collected from echocardiograms performed during hospitalization:

- Tricuspid annular plane systolic excursion (TAPSE): assessed using M-mode in the apical four-chamber view, expressed in millimeters (mm). ^14^

- Systolic pulmonary arterial pressure (sPAP): estimated by tricuspid regurgitation, expressed in millimeters of mercury (mm Hg), and inferred right atrial pressure, determined by the size and collapse of the inferior vena cava (IVC). The sPAP estimation was based on the peak tricuspideal regurgitation velocity (TRV), considering right atrial pressure (RAP) as described by the simplified Bernoulli equation. ^15^ RAP estimation was based on the diameter and respiratory variation in the diameter of the IVC: an IVC diameter ≤ 2.1 cm that collapses >50% with a sniff suggested a normal RAP of 3 mmHg, whereas an IVC diameter > 2.1 cm that collapses <50% with a sniff or < 20% on quiet inspiration suggested a high RAP of 15 mmHg. In scenarios in which the IVC diameter and collapse did not fit this paradigm, an intermediate value of 8 mmHg was used. ^16^

- Noninvasive RV-AP coupling: TAPSE/sPAP.

### Statistical Analysis

Continuous variables were reported as means or medians, with corresponding standard deviations and interquartile ranges, depending on their distribution. The Kolmogorov-Smirnov or Shapiro-Wilk test was used, depending on the sample size, to assess the normality of the distribution. For comparisons of continuous variables, Student’s t-test or the Mann-Whitney U test was employed, depending on the distribution. Categorical variables were presented as frequencies and percentages. Comparisons between proportions were conducted using the chi-square test or Fisher’s exact test, based on the expected value frequency.

The area under the curve (AUC) of the receiver operating characteristic (ROC) and the Youden index was used to determine the cutoff point with the highest sensitivity and specificity. Univariable Cox proportional hazards regression models were used to calculate hazard ratios (HR), as in a previous study. ^17^ Multivariable Cox regression was performed using covariates of clinical importance, as follows: age, gender, AF, renal dysfunction, and E/e’. Kaplan-Meier curves were generated, and survival differences were assessed based on the TAPSE/sPAP ratio.

Two-tailed p-values <0.05 were considered statistically significant. Statistical analysis was performed using SPSS software, Version 23.0 (IBM Corporation, Armonk, NY).

### Ethical Considerations

The study was approved by the institutional ethics and research committee and registered on the PRIISA.BA platform of the Ministry of Health of the City of Buenos Aires. At the time of hospitalization, patients provided consent for the transfer of personal data for scientific purposes. The study was conducted in accordance with national and international standards for the protection of research subjects, such as the Declaration of Helsinki, Resolution of the National Ministry of Health 1480/2011, City of Buenos Aires Law 3301, ANMAT Resolution 6677/10, and its amendments 4008 and 4009.

## Results

During the analyzed period, 465 patients were admitted for AHF with preserved or mildly reduced ejection fraction, of whom an echocardiogram and simultaneous measurement of TAPSE and sPAP could be performed in 361 patients (77%).

**Figure 1:**
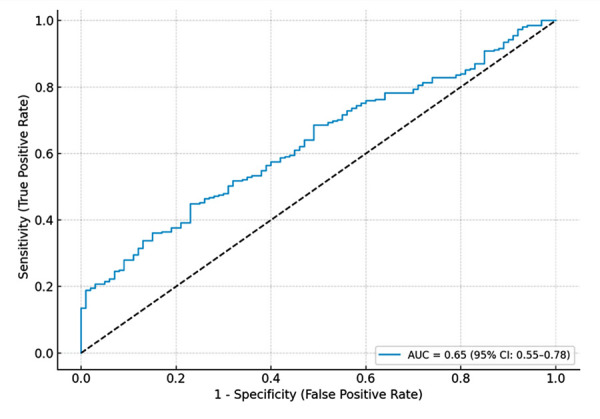
Receiver operating characteristic (ROC) curve of the TAPSE/sPAP ratio for predicting long-term all-cause mortality.

During a mean follow-up of 20.9 months, the primary outcome occurred in 100 patients (27.7%). The area under the curve (AUC) for the TAPSE/sPAP ratio in predicting all-cause mortality was 0.65 (95% confidence interval [CI]: 0.55-0.78), indicating moderate discriminative ability (Figure 1). 

**Figure 2:**
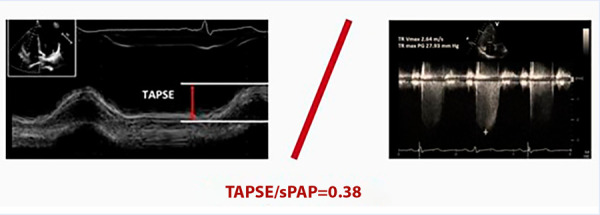
TAPSE/sPAP value for long-term mortality.

In relation to the primary endpoint of long-term mortality, it occurred in 20.5% of patients with a TAPSE/sPAP ratio ≥0.38 and 37.7% of those with a ratio <0.38 (Figure 2). The median TAPSE/sPAP among those who experienced the primary endpoint was 0.35 (IQR 0.23-0.5) and 0.45 (IQR 0.31-0.62) among those who did not (p<0.001).

sPAP: systolic pulmonary artery pressure. TAPSE: tricuspid annular plane systolic excursion.

The baseline characteristics between patients with and without reduced uncoupling were different; patients with a TAPSE/sPAP ratio <0.38 were more frequently women (60.9% vs. 43.8%, p=0.001) and had a history of hospitalization for heart failure (43.7% vs. 27.1%, p=0.001). No differences were found in age and comorbidities such as atrial fibrillation and chronic kidney disease. No differences were found between both groups in the triggering factors for decompensation, nor in the underlying disease. Upon admission, the clinical profile of patients differed; those with RV-PA uncoupling more frequently presented with a more severe AHF classification according to Society for Cardiovascular Angiography & Interventions (SCAI) criteria, as well as more frequently exhibited low cardiac output (7.9% vs. 1.4%) and less frequently had acute pulmonary edema (6.6% vs. 16.2%). The remaining baseline characteristics are detailed in Table 1.

**Table 1:** Basal characteristics according to TAPSE/sPAP

	TAPSE/sPAP ≥0.38 (n= 210)	TAPSE/sPAP <0.38 (n= 151)	p-value
Age in years (mean, SD)	78 (11)	76 (13)	0.2
Female sex (n, %)	92 (43.8)	92 (60.9)	0.001
Hypertension (n, %)	60 (28.6)	35 (23.2)	0.2
Dyslipidemia (n, %)	135 (65.2)	78 (51.7)	0.009
Diabetes (n, %)	60 (28.6)	35(23.2)	0.2
Atrial fibrillation (n, %)	29 (13.8)	27 (17,9)	0.2
Anemia (n, %)	42 (20)	30 (19.9)	0.2
Chronic pulmonary obstructive disease (n, %)	30 (14.3)	18 (11.9)	0.5
Chronic kidney disease (n, %)	39 (19.6)	28 (18.5)	0.9
Previous valvular surgery (n, %)	18 (8.6)	25 (16.6)	0.021
Previous myocardial infarction (n, %)	27 (12.9)	22 (14.6)	0.65
Previous AHF hospitalization (n, %)	57 (27.1)	66 (43.7)	0.001
Echocardiographic variables					
TAPSE in mm (median, IQR)	19 (15-22)	17 (14-20)	0.005
sPAP in mmHg (median, IQR)	40 (33-54)	45 (35-60)	0.011
TAPSE/sPAP (median, IQR)	0.45 (0.23-0.5)	0,35 (0.31-0.62)	<0.001

AHF: acute heart failure. CKD: chronic kidney disease. COPD: chronic obstructive pulmonary disease. IQR: interquartile range. SD: standard deviation. sPAP: systolic pulmonary artery pressure. TAPSE: tricuspid annular plane systolic excursion.

**Figure 3:**
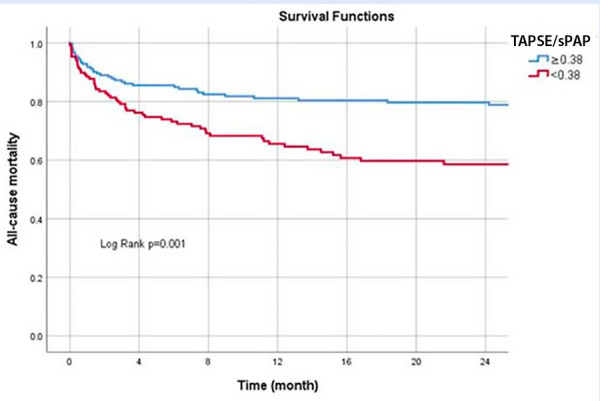
Kaplan-Meier for all-cause mortality according to TAPSE/sPAP.

The univariate analysis revealed that RV - PA uncoupling was associated with an increased risk of long-term mortality (hazard ratio [HR] 1.96 [95% CI, 1.32-2.92], P=0.001). Survival analysis (Kaplan-Meier) showed that patients with a TAPSE/sPAP ratio less than 0.38 had a higher risk of all-cause mortality **(Figure 3**). In the multivariate analysis, RV-PA uncoupling was associated with an increased risk of long-term mortality independent of age, sex, atrial fibrillation, renal failure, and E/e’ (HR 2.21 [95% CI, 1.26-3.81], P=0.005) (Table 2).

sPAP: systolic pulmonary artery pressure. TAPSE: tricuspid annular plane systolic excursion.

**Table 2:** Multivariate analysis in the prediction of all-cause long-term mortality

Variable	Hazard ratio	95% confidence interval	p-value
Age (per 1 year)	1.03	1.005-1.069	0.02
Female sex	1.15	0.66-2.01	0.61
Chronic kidney disease	0.84	0.42-1.66	0.62
Atrial fibrillation	0.67	0.26-1.72	0.41
E/E’ (per 1 unit)	0.99	0.94-1.04	0.7
TAPSE/sPAP <0.38	2.2	1.23-3.81	0.005

E/E’: ratio of early mitral inflow velocity to mitral annular early diastolic velocity. sPAP: systolic pulmonary artery pressure. TAPSE: tricuspid annular plane systolic excursion. CI: confidence interval.

## Discussion

Our study demonstrated that RV-PA uncoupling, as estimated by the TAPSE/sPAP ratio, was associated with increased mid- and long-term mortality in patients hospitalized for AHF with preserved and mildly reduced ejection fraction. This finding holds significant utility in predicting the prognosis of such a heterogeneous condition as HFpEF and HFmrEF. Furthermore, this association remained independent of risk factors such as age, sex, atrial fibrillation, renal failure, and diastolic dysfunction, underscoring the clinical relevance of right ventricular function in these patients.

HFpEF represents a heterogeneous syndrome influenced by various etiologic and pathophysiologic factors. Understanding that HFpEF is not a singular disease but rather a complex clinical syndrome is crucial. It exhibits diverse clinical manifestations, a substantial burden of comorbidities, and systemic pathophysiology affecting multiple organs. ^18^

The clinical phenotypes of heart failure with preserved ejection fraction (HFpEF) encompass diverse presentations: 1) “garden variety” HFpEF, often associated with hypertension, obesity, diabetes/metabolic syndrome, and/or chronic kidney disease; 2) CAD-associated HFpEF, characterized by multi-vessel coronary artery disease (CAD) as a primary driver of the HFpEF syndrome; 3) atrial fibrillation-predominant HFpEF, where uncontrolled atrial fibrillation is a key factor contributing to HFpEF syndrome progression; 4) right heart failure-predominant HFpEF, featuring pulmonary venous hypertension, occasionally with superimposed pulmonary arterial hypertension, and right ventricular dysfunction as the primary drivers of the clinical course; 5) hypertrophic cardiomyopathy-induced or hypertrophic-cardiomyopathy-like HFpEF, characterized by small left ventricular cavities with thick walls and a favorable response to negative inotropes; 6) multi-valvular HFpEF, marked by the presence of two or more moderate valvular lesions that contribute to HFpEF, often alongside other risk factors and etiologies; and 7) restrictive cardiomyopathies such as cardiac amyloidosis. ^19^

Over time, HFpEF was commonly perceived as a condition primarily affecting the left ventricle, characterized by a hypertrophic, noncompliant left ventricle leading to left atrial dysfunction and pulmonary congestion. However, recent research has shed light on the significant role of right heart disease in HFpEF pathogenesis, challenging this traditional view. ^20^ Understanding the importance of RV function and its interaction with the PA is crucial in HF patients, as they significantly influence prognosis. ^21^^-^^23^ The RV, intricately connected to the pulmonary circulation, undergoes dynamic hemodynamic changes during HF, affecting its contractility, compliance, and overall performance. Recognizing the intricate interplay between RV function and the hemodynamic burden imposed by the PA emerges as a pivotal aspect for predicting clinical outcomes and tailoring HF management strategies. ^24^^,^^25^

Our patient cohort represented a population of older adults, with a significant proportion of women and a high prevalence of multiple comorbidities. The results of our study complement earlier investigations and are consistent with recent studies emphasizing the prognostic significance of the TAPSE/sPAP ratio in AHF, establishing a strong correlation between it and the risk of mortality, hospitalization rates, and exercise capacity. ^26^^,^^27^ In a recently published study, ^28^ the prognostic value of the TAPSE/sPAP was investigated in patients hospitalized for AHF. A cohort of 333 patients from 39 French cardiology departments was assessed, with TAPSE/sPAP measured within 24 hours of admission. In-hospital major adverse cardiovascular events (MACEs) occurred in 15% of patients, with a TAPSE/sPAP threshold of <0.40 mm/mmHg identified as predictive. This ratio was independently associated with in-hospital MACEs even after adjustments for comorbidities and clinical severity. In contrast to our study, this investigation assessed the same population but focused on intrahospital outcomes.

Bok *et al.* also explored the prognostic significance of the TAPSE/sPAP ratio in patients with AHF. A TAPSE/sPAP ratio < 0.33 emerged as a predictor of increased mortality risk, even after adjustment for other variables. ^29^ This study had a similar follow-up period to our investigation and a comparable long-term all-cause mortality rate (33% vs. 27.7%).

However, the available information regarding the associations between RV-PA uncoupling and adverse outcomes in acute decompensated HFpEF and HFmrEF patients is more limited. The article by Nakagawa *et al*. also provides insights into the prognostic significance of RV-PA uncoupling in this group of patients. They found that the TAPSE/sPAP ratio <0.48 had a significant association with RV-PA uncoupling and adverse outcomes, including all-cause death, HF rehospitalization, and cerebrovascular events. This cutoff point was slightly higher than previously reported ratios. ^17^ In contrast to this study, our primary endpoint was long-term mortality, and additionally, the follow-up period was longer. We found that the cutoff point for predicting mortality was 0.38, which closely aligns with values reported in the literature ranging from 0.33 to 0.40, ^(6, 10)^ indicating the method’s reliability.

Our study has certain important limitations to mention. It is a retrospective analysis with the biases inherent in this type of design. However, the analysis of a prospectively loaded database, avoiding missing data and with a hard endpoint of interest such as the mortality assessed in all patients, could reduce potential biases. Additionally, we were unable to estimate either TAPSE or sPAP in 30% of our patients, with a significant portion of this percentage attributed to the lack of a systematic search for these parameters rather than technical difficulties in obtaining them. Another limitation of our study is the inability to distinguish between cardiovascular and non-cardiovascular causes of death during follow-up. Given the retrospective nature of the study and the lack of complete data regarding the specific cause of death in all cases, we were unable to perform a competing risk or subgroup analysis according to etiology. This is particularly relevant in this population, which is characterized by a high burden of comorbidities and a non-negligible proportion of non-cardiovascular mortality. Future prospective studies with detailed adjudication of mortality causes are warranted to better explore the prognostic value of the TAPSE/sPAP ratio in relation to cardiovascular vs. non-cardiovascular death. Finally, the study was carried out in a highly complex cardiovascular single center; therefore, the sample may not be representative of other centers. Despite these limitations, our findings are consistent with those reported in the literature.

This investigation shows that the utilization of a simple echocardiographic parameter like the TAPSE/sPAP ratio offers a powerful means to identify high-risk individuals, enabling early detection of adverse events during follow-up. This ratio serves as an invaluable non-invasive marker, providing clinicians with a comprehensive understanding of RV function and its intricate relationship with PA dynamics across various hemodynamic states. Its ability to offer crucial long-term prognostic insights emphasizes the importance of incorporating this parameter into routine clinical practice. These findings underscore the necessity for intensified monitoring and tailored heart failure-specific care strategies for patients identified as high-risk based on TAPSE/sPAP ratio measurements, ultimately aiming to improve patient outcomes and enhance the overall management of HFpEF and HFmrEF patients.

In conclusion, in patients hospitalized for AHF with preserved and mildly reduced ejection fraction, RV-PA uncoupling as determined by the TAPSE/sPAP ratio was independently associated with long-term all-cause mortality. This finding underscores the clinical significance of this simple echocardiographic parameter in identifying individuals at heightened risk for adverse events during follow-up. Further investigation into the clinical implications is warranted to optimize patient management and improve outcomes in this AHF population.
